# Perceptual adaptation to dysarthric speech is modulated by concurrent phonological processing: A dual task study

**DOI:** 10.1121/10.0035883

**Published:** 2025-03-10

**Authors:** Patti Adank, Han Wang, Taylor Hepworth, Stephanie A. Borrie

**Affiliations:** 1 Department of Speech, Hearing and Phonetic Sciences, University College London, London, United Kingdom; 2 Clinical Systems Neuroscience Section, Department of Developmental Neurosciences, Great Ormond Street Institute of Child Health, University College London, London, United Kingdom; 3 Department of Neurosurgery, Great Ormond Street Hospital for Children, National Health Service (NHS) Foundation Trust, London, United Kingdom; 4 Department of Communicative Disorders and Deaf Education, Utah State University, Logan, Utah 84322, USA

## Abstract

Listeners can adapt to noise-vocoded speech under divided attention using a dual task design [Wang, Chen, Yan, McGettigan, Rosen, and Adank, Trends Hear. **27**, 23312165231192297 (2023)]. Adaptation to noise-vocoded speech, an artificial degradation, was largely unaffected for domain-general (visuomotor) and domain-specific (semantic or phonological) dual tasks. The study by Wang *et al.* was replicated in an online between-subject experiment with 4 conditions (*N* = 192) using 40 dysarthric sentences, a natural, real-world variation of the speech signal listeners can adapt to, to provide a closer test of the role of attention in adaptation. Participants completed a speech-only task (control) or a dual task, aiming to recruit domain-specific (phonological or lexical) or domain-general (visual) attentional processes. The results showed initial suppression of adaptation in the phonological condition during the first ten trials in addition to poorer overall speech comprehension compared to the speech-only, lexical, and visuomotor conditions. Yet, as there was no difference in the rate of adaptation across the 40 trials for the 4 conditions, it was concluded that perceptual adaptation to dysarthric speech could occur under divided attention, and it seems likely that adaptation is an automatic cognitive process that can occur under load.

## I. INTRODUCTION

Everyday listening often involves exposure to speech signals that are distorted and/or unfamiliar such as noise-vocoded speech (Huyck and Johnsrude, 2012; Wang *et al.*, 2023). Generally, on first hearing distorted or unfamiliar speech, perception performance deteriorates compared to clear or familiar speech but rapidly improves after short-term exposure to a few distorted or unfamiliar sentences or phrases as a result of *perceptual adaptation*. Perceptual adaptation can be defined as a fast and short-term dynamic improvement in speech perception performance. Perceptual adaptation is a type of *perceptual learning* of speech, which generally refers to long-term changes to our speech perception processing system. Whereas the two concepts, perceptual adaptation and perceptual learning, have been used interchangeably to refer to longer and short-term perceptual changes and processes (Banks *et al.*, 2015; Davis *et al.*, 2005; Hervais-Adelman *et al.*, 2008), here, we will use the term adaptation as our study focuses on short-term improvements as to the recognition of unfamiliar speech input.

Perceptual adaptation for speech is a well-established phenomenon and it has been repeatedly shown that listeners, after only a handful of sentences (Cooke *et al.*, 2022), improve their recognition of a wide variety of variation types in speech, including speech in an unfamiliar accent (Baese-Berk *et al.*, 2013; Banks *et al.*, 2015; Bradlow and Bent, 2008; Clarke and Garrett, 2004), noise-vocoded speech (an artificial distortion in which all harmonic information has been replaced with broadband noise, cf. Shannon *et al.*, 1995; Davis *et al.*, 2005; Hervais-Adelman *et al.*, 2008; Huyck and Johnsrude, 2012; Wang *et al.*, 2023), fast speech (Adank and Janse, 2009; Dupoux and Green, 1997; Sebastián-Gallés *et al.*, 2000), and dysarthric speech (Borrie *et al.*, 2017; Borrie and Lansford, 2021; Borrie and Schäfer, 2015; Liss *et al.*, 2002). Perceptual adaptation of speech is generally studied by exposing participants who are naive to the type of variation to a series of words/sentences displaying that variation and asking them to perform a comprehension task (e.g., a speeded semantic verification task as in Adank and Devlin, 2010; Kennedy-Higgins *et al.*, 2020; or a word or sentence recall task, as in Cooke *et al.*, 2022; Trotter *et al.*, 2021; Wang *et al.*, 2023). Although perceptual adaptation for speech has been studied extensively (Bent and Baese-Berk, 2021; Borrie *et al.*, 2012a; Samuel and Kraljic, 2009), relatively little is known about the cognitive mechanisms mediating this form of implicit learning.

Moreover, in cognitive psychology, the relationship between attention and perceptual adaptation/learning has been formalised in several models for information processing and perceptual learning, including Goldstone (1998), the predictive coding theory (Friston and Kiebel, 2009), and Amitay's reverse hierarchy theory (RHT; Ahissar and Hochstein, 2004; Amitay, 2009). These models converge on the idea that attention exerts a top-down influence on perception by selectively amplifying the salience of certain low-level sensory cues over time, effectively guiding perceptual adaptation and learning. Goldstone and RHT propose that attentional processes play a crucial role in refining sensory representations by shifting focus from task-irrelevant to task-relevant cues. Such attentional reallocation enables observers to prioritise task-relevant features at the expense of task-irrelevant features, leading to the development of more precise and efficient low-level representations. Goldstone highlighted this mechanism as integral to adaptive changes in perception, whereas RHT emphasised the hierarchical nature of this process, suggesting that higher-level attentional processing reweight sensory inputs. The predictive coding theory conceptualised attention as a computational process that integrates top-down predictions with bottom-up sensory input. In this framework, attention also filters and enhances relevant sensory information but also facilitates resolution of prediction errors, thereby optimising perceptual learning and adaptation. This integration allows the perceptual system to continuously refine its internal models of the environment based on incoming sensory data.

The frameworks discussed above, thus, identify attention as a requirement for perceptual learning and adaptation to occur but are underspecified as to how attentional cognitive resources are employed. This lack of specification may be, first, due to the fact that, conceptually, attention is not easy to characterise, and there exists disagreement on its definition and its component cognitive processes (Hommel *et al.*, 2019; Krauzlis *et al.*, 2023). As a detailed discussion of attention is outside the scope of this paper, we will pragmatically conceptualise “attention” as the engagement of cognitive process(es) that allow us to successfully complete a behavioural task. Second, these theoretical frameworks appear agnostic of the subclassification of attentional resources, depending on the need to focus on a single or concurrent tasks (Mattys and Palmer, 2015). Mattys and Palmer (2015) identify *focused attention* as a situation in which participants pay attention to one stimulus only, whereas *selective attention* involves ignoring a concurrent stimulus, and *divided attention* is identified as a situation in which participants are required to process the target and concurrent stimulus. It is, thus, necessary to clarify whether attentional resources employed during perceptual adaptation are focused, selective, or divided.

It is important to clarify how a cognitive process, such as perceptual adaptation, relies on attentional resources as it can help determine whether this process is predominantly controlled or automatic in nature. Cognitive processes have been characterised as residing somewhere on a continuum between automatic or controlled (also referred to as type1/type 2 processes; Melnikoff and Bargh, 2018). Automatic processes are thought to be efficient, unintentional, uncontrollable, and unconscious. Examples of (partial) automatic processes are touch-typing or driving. Controlled processes, on the other hand, have been characterised as inefficient, intentional, controllable, and conscious. Examples of controlled processes involve decision-making and problem-solving.

A key difference between an efficient and a controlled process depends on its reliance on attentional resources. A process can be considered to be *efficient* if it persists under cognitive load, i.e., a satiation where attention is divided instead of focused/selective, such as the requirement to perform a concurrent task while adapting to degraded speech. An efficient behaviour can be performed while central processing resources are occupied with a concurrent task, for example, driving while maintaining a conversation. To determine whether perceptual learning of degraded speech is *efficient*, it needs to be considered as to which extent this process is resistant to cognitive load and reliant on attentional resources.

Several studies aimed to elucidate the role of focused, selective, and divided attention to natural (foreign accent) and artificial (noise-vocoding) variation in speech perception using a dual task paradigm (Hunter and Pisoni, 2018; Huyck and Johnsrude, 2012; Wang *et al.*, 2023; Wild *et al.*, 2012). In a dual task paradigm, participants perform a primary speech perception task plus a secondary task. The aim of this secondary (dual/concurrent) task is to place an additional load on the speech perception system to learn more about the cognitive processes involved in perceptual adaptation. Several studies have used the dual task paradigm to show that perception of artificially degraded speech is affected by the increased load imposed by the dual task (Hunter and Pisoni, 2018; Wang *et al.*, 2023). For instance, participants in Hunter and Pisoni (2018) listened to noise-vocoded speech and reported key words. Participants also completed a dual task consisting of a low-load (three digits) or high-load (seven digit) recall task. They observed that word report was less accurate for a high-load than for the low-load dual task, thus, demonstrating that the role of divided attention in perceiving noise-vocoded speech can be established using a dual task paradigm. Huyck and Johnsrude (2012) demonstrated that focused attention not only modulates the perception of noise-vocoded speech but also the perceptual adaptation to this type of speech. Participants in one group attended to noise-vocoded sentences and repeated back the words that they heard while another group selectively attended to concurrent auditory bursts or visual ellipses and decided whether a target pattern was presented. Before and after training, all participants conducted a testing phase during which they completed the speech task. Perceptual adaptation to noise-vocoded speech only occurred when attention was directed to the speech task rather than the concurring auditory and visual distractors, suggesting a key role of attention in perceptual adaptation of speech. However, as the speech and dual task were not concurrent, the results only apply to situations in which attention is entirely focused on either the speech signal or the other task. Thus, the results of Huyck and Johnsrude show the effect of focused attention, and not of selective or divided attention, on perceptual adaptation of speech.

Wang *et al.* (2023) extended the study by Huyck and Johnsrude by evaluating how perceptual adaptation to speech occurs under divided attention. Participants in Wang *et al.* were asked to split their attention between the primary speech perception task and a dual task. In experiment 1, 192 participants in 4 groups in an online setup completed a word recall task for 40 Bamford-Kowal-Bench (BKB) corpus sentences (Bench *et al.*, 1979). All sentences were six-band noise-vocoded. One group (single task) only completed the speech perception task (control) while the other three groups also completed a dual visuospatial decision task. Participants judged the orientation of a Gabor patch (Calder-Travis and Ma, 2020), depicting a sine wave grating. Each Gabor patch was located on the circumference of an imaginary circle placed in the centre of the screen. Participants were asked to judge whether the orientation of the Gabor patch was equal to 45°. Half of the patches were presented at 45°. The visuomotor task had three difficulty levels (dual easy, dual intermediate, and dual hard), and each of the three dual task groups completed the task at a single difficulty level. All participants adapted to the noise-vocoded sentences as primary performance increased between 7% and 24% over the course of the experiment. Participants adapted the most in the dual hard group (29%), which showed the poorest task performance at the start of the experiment. At the end of the experiment, all groups reached the same overall level of performance on the speech task (65% correct). Participants all scored above chance level (50%) on the dual tasks (dual easy, 83%; dual intermediate, 77%; and dual hard, 61%). These results showed that sentence recognition improved at least as much as in the single speech task, meaning that perceptual adaptation to noise-vocoded speech can occur under divided attention. The time course of perceptual adaptation was modulated by the difficulty of the dual task: compared to the single speech task, participants showed more perceptual adaptation in the beginning of the experiment.

In experiment 2, Wang *et al.* established whether the attentional processes supporting perceptual adaptation are domain-general or domain-specific (e.g., phonological or lexical) in nature. Domain-general attentional mechanisms allocate attention to general cognitive processes that are associated with nonlinguistic processes or tasks, such as decision-making or visual processing (e.g., attribution of visual attention to a depicted shape or form). In the context of perceptual adaptation, domain-specific mechanisms allocate attention to processes or tasks involved in spoken language recognition, such as segmental/suprasegmental or phonological processes (e.g., deciding on the first phoneme of a word or deciding how many syllables a word contains), or word-level or lexical processes or tasks (e.g., deciding whether a word is man-made or not). Wang *et al.* included a domain-specific task to evaluate the effect of exhausting phonological or lexical attentional resources on perceptual adaptation to noise-vocoded speech. The experiment, again, tested 192 participants in 4 groups. The single group results were included from experiment 1, and 144 new participants were tested across 3 groups. Participants were shown the same set of words across the three groups. The words were two or three syllables long and referred to either an animal (e.g., *penguin*) or object (e.g., *lipstick*). All words were presented at an angle on the screen as in experiment 1, replacing the Gabor patches to enable the use of the same stimuli in all groups. Participants in the phonological condition judged if the word consisted of two or three syllables. In the lexical condition, participants judged if the word represented an object or an animal. In the visuomotor condition, participants judged if the word was presented at a 45° angle. The results showed that participants all adapted to the noise-vocoded sentences, showing 14%–23% improvement. There was no difference in the pattern of perceptual adaptation across the four groups, demonstrating that adaptation to noise-vocoded speech engages domain-general processes and does not rely on domain-specific attentional processes.

The results of Wang *et al.* are noteworthy in two ways. First, they have implications for the predictive coding framework of perceptual learning (Friston and Kiebel, 2009) as they suggest that divided attentional resources are sufficient for successful adaptation. Second, their results have implications regarding the nature of perceptual learning as a cognitive process, supporting the notion of perceptual adaptation as an efficient—and, therefore, largely automatic—cognitive process. However, below, we argue that a further test of the role of attentional resources may be needed before it can be concluded that divided attention is sufficient for adaptation to occur.

### A. The current study: Adapting to a natural degradation

Wang *et al.* used an artificial degradation of the speech signal, namely, noise-vocoded speech, which is created by, first, extracting amplitude envelopes from a number of frequency bands (typically between 1 and 32; McGettigan *et al.*, 2014) of the acoustic signal to modulate the corresponding bands of a noise carrier signal. A noise-vocoder removes spectral detail while preserving low-frequency amplitude and temporal information. In normal-hearing listeners, the intelligibility of the speech signal increases logarithmically with the number of bands, meaning that performance over bands increases. The vocoder removes all harmonic details from the signal, including most aspects of the speaker's voice, and the distortion, therefore, results in a low-level, acoustic degradation likely affecting speech processing mostly at a pre-lexical, auditory, level.

The current study explores the possibility that for perceptual adaptation to be affected by a dual task, this dual task should occupy attentional resources at the same linguistic processing level as the degradation of the speech signal. We will focus specifically on a match between the primary speech task and dual task at the segmental/suprasegmental level by replacing the artificial distortion used in Wang *et al.*, with naturally occurring variation in speech, namely, dysarthric speech.

We replicated the study by Wang *et al.* using the four task conditions from their experiment 2 (single, visual, phonological, and lexical), using a natural, noncanonical type of speech that is more likely to display segmental/suprasegmental variation than noise-vocoded speech and, thus, more likely to engage linguistic processes at the same processing level as the phonological task. Dysarthria is a motor speech disorder arising from neurological origins, including stroke, brain injury, or degenerative diseases such as Parkinson's disease or amyotrophic lateral sclerosis. This natural degradation will, therefore, provide a closer test of the reliance of the perceptual system on attentional resources during perceptual adaptation to speech under cognitive load. Using a natural degradation, we will thereby be able to confirm, more conclusively, perceptual adaptation to degraded speech as an efficient and, hence, largely automatic cognitive process. Further, the use of dysarthric speech allows theoretical models to extend to real-world degradation, where the acoustic distortions are not entirely predictable but rather vary in systematic and nonsystematic ways (Borrie *et al.*, 2012a). Beyond theoretical implications, the study of perceptual adaptation of dysarthric speech is of high clinical import. Traditional treatments to improve intelligibility of dysarthria have focused on the person with the speech disorder (e.g., talk louder or speak more clearly). However, more recently, a body of literature has shown statistically significant and clinically meaningful intelligibility gains when the weight of behavioural change is shifted from the speaker to the listener through perceptual adaptation to dysarthric speech (see Borrie and Lansford, 2021, for a review).

We replaced noise-vocoded speech with speech produced by someone with dysarthria. The dysarthric speech signal is characterised by segmental and suprasegmental degradations, which typically reduce intelligibility of the speech signal. A large body of work has shown that listeners can perceptually adapt to dysarthric speech after limited exposure (see Borrie and Lansford, 2021, for a comprehensive review). The intelligibility increase for dysarthric speech is accompanied by enhanced processing of segmental information as demonstrated by improvements in percent syllable resemblance (Borrie *et al.*, 2012b, 2013), substitution errors (Spitzer *et al.*, 2000), and consonant identification. Additionally, following exposure, listeners are also better able to process suprasegmental information, reflected in more reliable use of syllabic stress cues to decipher dysarthric speech (Borrie *et al.*, 2012a; Borrie *et al.*, 2012b, 2013). Thus, perceptual adaptation to dysarthric speech is driven by adaptation to segmental and suprasegmental regularities. However, it should be noted that the variation in dysarthric speech is not entirely phonological in nature. While this type of speech displays segmental variation at phonetic and phonological levels, it also contains suprasegmental variation that is not captured by the phonological dual task, hence, there is no perfect match between the type of variation in the speech signal and the phonological dual task.

Compared to other types of speech that display variation/degradation at phonological levels, such as time-compressed speech (Dupoux and Green, 1997), natural fast speech (Adank and Janse, 2009), and speech in an unfamiliar native accent (Adank *et al.*, 2009; Bradlow and Bent, 2008; Floccia *et al.*, 2009), dysarthric speech was the best option for our experimental purposes. Time-compressed speech is an artificial distortion that is thought to display phonological variation, and listeners can adapt to speech that has been compression to ∼30% of its original duration (Sebastián-Gallés *et al.*, 2000). Yet, time-compressed speech contains an intrinsic confound between duration and the presence of phonological duration. Using a very short speech signal would pose challenges for our design and timing of the presentation of the stimulus in the dual task. Natural fast speech, such as dysarthric speech, displays segmental plus suprasegmental variation but suffers additionally from the same timing confound as time-compressed speech. Finally, accented speech contains segmental plus suprasegmental variation and also poses challenges on the side of the listener as listeners must be unfamiliar with the accent. As we used an online design, it was challenging to control for the United States (U.S.) listeners' specific accent. We, therefore, decided that using dysarthric speech would be a fair compromise between the type of segmental/suprasegmental variation in the signal and requirements of our online design.

### B. Aim and hypotheses

Our aim was to establish whether perceptual adaptation to a natural distortion—dysarthric speech—can occur under divided attention to gain further insight into the nature of perceptual adaptation as a largely automatic or controlled cognitive process. We evaluated the rate and shape of perceptual adaptation after exposing listeners to a dual task that engaged comparable mechanisms needed to identify and extract relevant cues for mapping the unfamiliar signal onto existing mental speech. Hypothesis 1 states that if perceptual adaptation can occur under divided attention, participants will adapt at a similar rate and follow the same pattern in all four conditions. Hypothesis 2 states that if adaptation to a natural degradation in speech that includes phonological variation requires attention, listeners will adapt less and/or follow a different pattern for the phonological condition than for the other three conditions.

## II. METHOD

### A. Participants

We determined our sample size based on Wang *et al.* and, thus, a total of 192 participants [96 female (F) and 96 male (M) between 18 and 35 years of age (*Y*), mean = *Y*, standard deviation (SD) = 5.2*Y*] completed the online experiment. All were self-declared to be monolingual American English speakers residing in the U.S. at the time of the experiment. All reported normal hearing and normal or corrected-to-normal vision and no neurological disorders (including dyslexia). Participants were assigned to one of four conditions (*n* = 48 per condition). The demographics per condition were similar: single-task condition (24 F, mean = 25.7*Y*, SD = 5.2*Y*), dual visual condition (24 F, mean = 26.8*Y*, SD = 5.6*Y*), dual phonological condition 24 F, mean = 25.5*Y*, SD = 4.9*Y*), dual lexical condition 24 F, mean = 27.1*Y*, SD = 5.2*Y*). We based the sample size per condition on our recent online study by Wang *et al.*, which investigated perceptual adaptation to noise-vocoded speech in a between-group design. After collecting an initial 192 participants, we conducted a post-experiment screening and recruited new participants to replace: (1) 7 participants whose performances (see Sec. II D) in the speech or dual task were > 3 SDs away from the group mean; (2) 19 participants whose response accuracy in the dual task was below chance level (i.e., <50% correct); (3) 4 participants who performed the task in a noisy environment; (4) 2 participants who produced low-quality or inaudible speech responses; and (5) 3 participants who had a non-native English accent. All participants in the final data set passed post-experimental screening, and were recruited via the online recruitment platform Prolific (Peer *et al.*, 2017) and paid at a rate corresponding to £9.00 per hour. The experiment was approved by the Research Ethics Committee of University College London (UREC, No. 0599.001).

### B. Materials

#### 1. Speech task

Speech stimuli consisted of previously collected audio recording of 80 syntactically plausible but semantically anomalous phrases (e.g., “*amend estate approach*,” cf. Table III in the Appendix) elicited from a male American English native speaker with dysarthria. The phrases, modeled on the original work of Cutler and Butterfield (1992), were created specifically to examine speech recognition in adverse conditions (Liss *et al.*, 1998). Phrases were all six syllables in length and ranged from three to five words. From the original set of 80 phrases, 42 were drawn. Each word was unique across the 42 phrases, except that we allowed function words, including *the*, *and*, *but*, *or*, *for*, *a*, *an*, *to*, *in*, *with*, and *can* to duplicate across sentences. The phrases, which restrict top-down processes associated with sematic content while emphasising bottom-up processing associated with acoustic input, have been frequently used in studies examining the perception of dysarthric speech (Borrie *et al.*, 2012b; Borrie and Lansford, 2021).

The speaker with dysarthria presented with a moderate hypokinetic dysarthria secondary to Parkinson's disease. Dysarthria diagnoses and severity ratings were made by three certified speech-language pathologists with expertise in assessment and diagnosis of motor speech disorders. The speech of the talker with Parkinson's disease represented a classic hypokinetic dysarthria, characterised perceptually by imprecise articulation, variable speech rate, short rushes of speech, monotone, mono-loudness, and a breathy voice.

Phrase productions were recorded via a cardioid lavalier microphone positioned approximately 20 cm from the speaker's mouth. Speech was recorded directly to a laptop using Adobe audition (San Jose, CA), via a Shure X2U XLR-to-USB signal adapter (Niles, IL) with a sampling rate of 48 kHz and 16 bits of quantization. After recording, each phrase had its average intensity scaled to 70 dB sound pressure level (SPL) for consistency in the listening experiments.

#### 2. Dual tasks

The dual tasks were similar to the phonological, semantic, and visuomotor dual tasks used in Wang *et al.* Stimuli for the tasks were a set of words taken from SUBTLEX-UK, a word frequency database of British English based on television subtitles (Van Heuven *et al.*, 2014). It was necessary to select new words for the dual tasks as the sentence material of the primary speech task differed from Wang *et al.* We extracted nouns of medium- to high-frequency use (which have a Zipf measure of 3–4.5; see van Heuven *et al.*, 2014) and further selected two-syllable and three-syllable words that referred to an animal or a man-made object (e.g., *leopard*, *kangaroo*, *boiler*, and *camera*). The final stimulus set contained 42 words—40 for main trials and 2 for familiarisation. The 40 main trial words were balanced for their syllable counts and semantic category. We used ten words for each of these four subsets: two-syllable animal, two-syllable objects, three-syllable animal, and three-syllable objects.

**FIG. 1.**
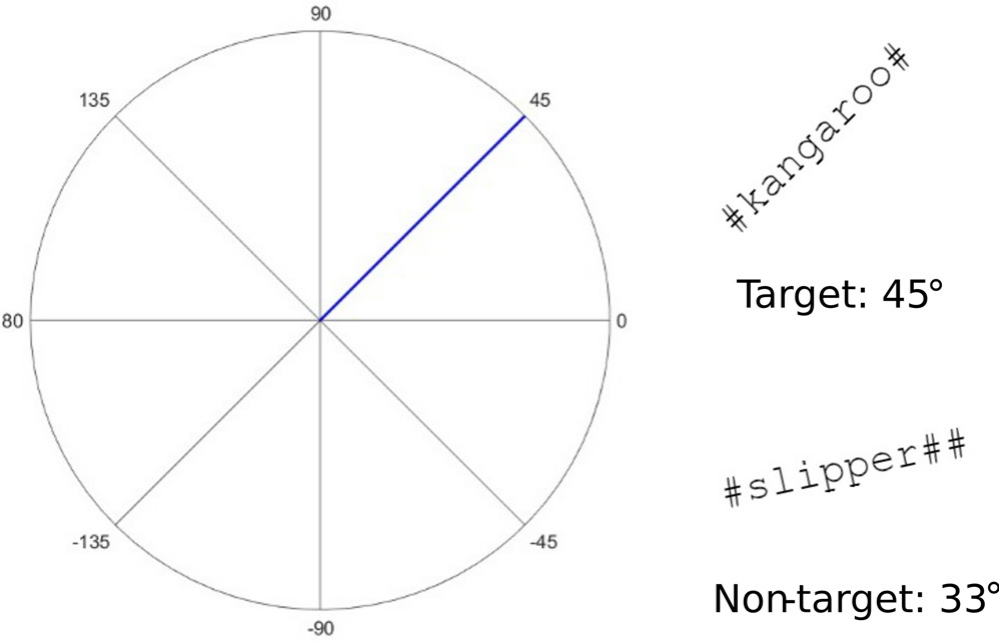
The example words displayed here are a 45°, three-syllable animal (i.e., kangaroo) and a non-45°, two-syllable manmade object (i.e., slipper). The orientation of a visual word is defined as the angle formed by the horizontal axis and the patch. The target orientation (45° clockwise from vertical) is highlighted as the blue line on the coordinates. For illustration purposes, the plots of words are, thus, not scaled to their actual sizes. These examples do not exhaust all possible orientations of a nontarget word.

The stimuli were presented as visual words on the participant's monitor. The stimulus in each trial was a word in a black font (height = 0.65 cm) displayed on a white background. Following Wang *et al.*, we further manipulated the orientation of these words for the visual task—half were 45° clockwise from vertical (target) and the other half were 24° < Δ ≤ 36° apart from 45° (Δ; nontarget; see Fig. 1). The orientations of the nontarget words came from a uniform distribution such that all possible nontarget orientations were equally likely to enter the sample. Furthermore, the number of 45° words was counterbalanced across the four subsets of ten stimuli, differing in their syllable counts and semantic category. Thus, in each subset, five words were presented at 45° and five deviated from 45°. To prevent participants from estimating the number of syllables in a word from its visual length, we equated the visual length across stimuli by padding each word with hashtag (#) symbols and displayed in the monospaced font Courier New. Each word's location varied randomly across trials in the range of −9.4–9.4 cm horizontally from the centre of the monitor. As in Wang *et al.*, this measure was taken to prevent participants from using an auxiliary tool (e.g., a ruler) to judge the orientation of a word. The visual-word stimuli were generated using a matlab script (Natick, MA).

### C. Procedure

The experiment was hosted online in an online testing environment (Anwyl-Irvine *et al.*, 2020).^1^ Participants were first asked to report their monitor size before a customised JavaScript (Oracle Corporation, Austin, TX) detected their display resolution. Thirty-two participants who reported a monitor size smaller than 12.1 in. in diagonal or had a resolution less than 1440 × 900 were disqualified from participation because the horizontal dimension of a monitor smaller than 12.1 in. (with a typical 16:10 ratio) was shorter than that of the stimulus image (22.6 cm); hence, the visual stimuli would have been scaled to smaller than their desired size. Those who passed the display validation were provided further information about the experiment and asked to provide consent. They were requested to turn on the auto-play of audio and video and enable cookies online use.^1^ Participants were required to plug in their headphones and not use wireless (Bluetooth, Kirkland, WA) headphones. To exclude those who were not wearing headphones, participants passed a screening (Woods *et al.*, 2017). After passing this screening, they were presented with 1000 ms of white noise, which they could replay to adjust their volume to a comfortable level. The final validation was a microphone check, in which participants were asked to record their own voices to check if their responses could be recorded.

Before the main experiment phase, a customised JavaScript enabled full-screen mode and hid all window components of the browser (i.e., the tabs, address bar, and bookmark bar). Then, a tool, which is provided online,^1^ guided participants to calibrate their monitor such that the visual stimuli could be displayed at an equivalent size across all participants regardless of their monitor size and resolution. Participants were asked to place a standard-size credit card against an image of the card shown on the monitor and to drag a slider until the size of the image matched that of the physical card. The calibration programme then used the pixel (px) counts of the image to acquire the px density (px per in.) of the monitor and scaled the visual stimuli to the width (in px) corresponding to the desired size (in cm). Participants were then presented with a sample word that illustrated the target orientation for the stimuli of the dual task (Fig. 1).

**FIG. 2.**
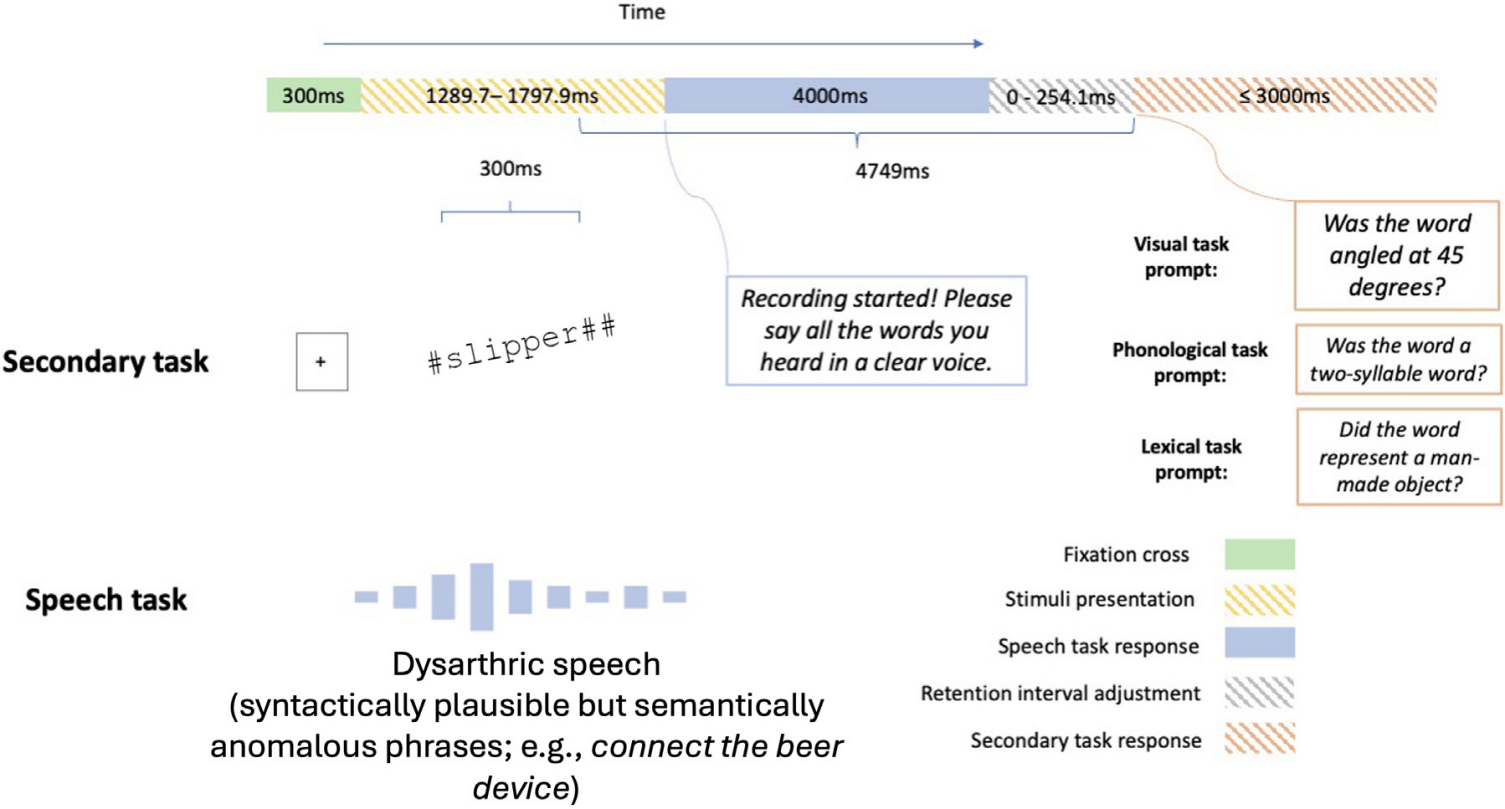
During the experiment, participants recognised a dysarthric phrase while they saw a visual word flashing briefly. All dual task conditions used the same set of stimuli. Participants in the visual task condition decided whether the word was oriented at 45° clockwise from vertical. Participants who did the phonological task judged whether the word (e.g., slipper) was a two-syllable word. Those who performed the lexical task decided whether the word (e.g., kangaroo) was a man-made object. A retention interval adjustment was added between the responses to the speech task and visual task such that the duration between the end of the visual-word presentation and the start of the dual task response was identical across trials. The plots of the fixation cross and the visual word are for illustration only and not scaled to actual size.

In the main experiment, participants in a between-group design recognised a dysarthric phrase while performing either a visual, phonological, or lexical dual task (Fig. 2). They performed two familiarisation trials before the 40 experimental trials. The procedure for the familiarisation and main trials was identical, except that the correct answer was revealed after the participant gave their responses. Participants were not instructed to prioritise either of the two tasks and only told to perform both tasks together as it would be hard to prevent participants from dynamically changing their allocations of resources over time, which might be particularly true for a real-life scenario. In each trial, a fixation cross was displayed at the screen's centre for 300 ms. They, then, heard a six-band BKB sentence plus a visual word presented for 300 ms. The word appeared 150 ms prior to the midpoint of the sentence duration and ended at 150 ms following the midpoint. Subsequently, participants were given 4 s to repeat back the sentence. Next, participants were prompted to report whether the word was angled at 45° (visual task), whether the word was a two-syllable word (phonological task), or whether the word represented a man-made object (lexical task). Participants had 3 s to respond by pressing the left (“yes”) or right (“no”) arrow key. Because sentences had different durations, a variable (0–254 ms) blank window was interleaved between the spoken and key press responses to ensure that each trial had the same retention interval (4749 ms) between the end of the visual-word presentation and the start of the dual task response window.

Participants in the single-task condition only heard and responded to the speech stimuli, and each trial terminated after the spoken response window. In the dual task, half of the trials had a correct answer of “yes”. For each participant, the trial order was randomised, yet, the pairing between a sentence and a visual word in a trial was the same. After the main experiment, participants completed a questionnaire, where they indicated how much effort and attention they had invested on a 0–100 scale (see Questionnaire 1 and Table VI in the Appendix for details and analysis). The experiment took 23 min (SD = 13.1 min) on average.

### D. Dependent measures

The percentage of correctly recognised key words for each sentence was the main dependent measure. Following Trotter *et al.* (2021) and Wang *et al.* (2023), words with incorrect suffixes (e.g., -s, -ed, and -ing) were scored as correct, but words (including compound words) reported in part (e.g., “raindrops” instead of “raincoats”) were scored as incorrect. All materials were double-coded, coders were blind to the stimulus condition, showed high inter-rater agreement (Cohen's kappa > 0.95), and trials with disagreement between raters were discussed and agreed upon. Trials without a response were coded as 0% correct. RTs in milliseconds and correctness (i.e., zero or one) on each trial were measured for the dual task to describe the change in performance over the whole task. Trials having an incorrect or no response for the dual task were excluded from the RT analysis. Finally, subjective ratings were collected from the questionnaire to describe the effort and attention that participants had invested in each of the dual tasks (see Questionnaire 1 in the Appendix).

### E. Analysis

We fit a set of generalised linear mixed-effect models (GLMMs) using the glmer( ) function in the lme4 *R*-package (version 1.1–27.1; Bates *et al.*, 2015) to uncover the relationship between the predictors and behavioural responses in the main experiment. We analysed the % correct data from the phrase recognition task and the correctness and RTs for the dual tasks. RT models assumed a gamma distribution of residuals and used a log-link function with a bobyqa optimiser to account for the skewed RT distributions (Lo and Andrews, 2015), whereas models for % correct and correctness assumed binomially distributed residuals and adopted a logit link function. All models had task (i.e., single, dual easy, dual intermediate, and dual hard for speech % correct and dual easy, dual intermediate, dual hard for visual task correctness and RTs), trial, and their interaction as predictors. Following Wang *et al.* (2023) and Cooke *et al.* (2022), a logarithmic transformation ( ${\text{log}}_{e}x$) was applied to trial to account for the logarithmic trend of rapid perceptual adaptation (i.e., greater accuracy and speed improvements in early trials).

The model for speech % correct initially included random intercepts for participant and sentence and random slopes for trial by participant and task by sentence. The maximal models for dual task correctness and RTs included random intercepts for participant and word prompt and random slopes for trial by participantand task by word prompt. To select an optimal fitting model for our data, we first removed random effects that caused a convergence failure (Mickan *et al.*, 2020). Next, we excluded the random effects whose inclusion yielded inaccurate estimates of the raw responses—a sign of over-fitting (Nannen, 2003). Last, we applied a backward model-selection procedure using the anova( ) function, which compared the goodness-of-fit (i.e., maximised log-likelihood) of two models given the data while penalising for the complexity of the models. Each time, we performed a comparison between a model and a simpler model, excluding a certain random effect and removing the effect where it did not significantly contribute to the model fit. We continued such comparisons until we found the best fitting model. The best fitting model for speech % correct included random intercepts for the participant and random slopes for trial by participant. The final model for dual task correctness included random intercepts for participant and prompt and random slopes for trial by participant. The model for dual task RT had random intercepts for prompt and random slopes for trial by prompt. Finally, pair-wise comparisons (with Holm–Bonferroni correction for *p* values) were conducted for the best fitting models using the pairs( ) function in the emmeans *R*-package (version 1.10.0; Lenth *et al.*, 2024) to estimate the difference in the mean accuracy and RTs across task conditions.

Our previous findings have demonstrated that the magnitude of perceptual adaptation under divided attention tends to be subtle (i.e., ∼10%; Wang *et al.*, 2023), therefore, the adaptation differences across task conditions might be diluted over the course of 40 trials, leading to a nonsignificant interaction between trial and task. To this end, the first derivatives of the estimated performance measures from the final GLMM model with regard to trial were calculated using the base *R* diff( ) function [i.e., first_derivative = diff(estimated_performance)/diff(trial)] to describe the rate of change in each condition. A separate GLMM was then fit to the speech % correct data from the rapid phase of adaptation (i.e., the first ten trials; also see Fig. X2) to examine the trial × task interaction.

## III. RESULTS

### A. Speech task

**FIG. 3.**
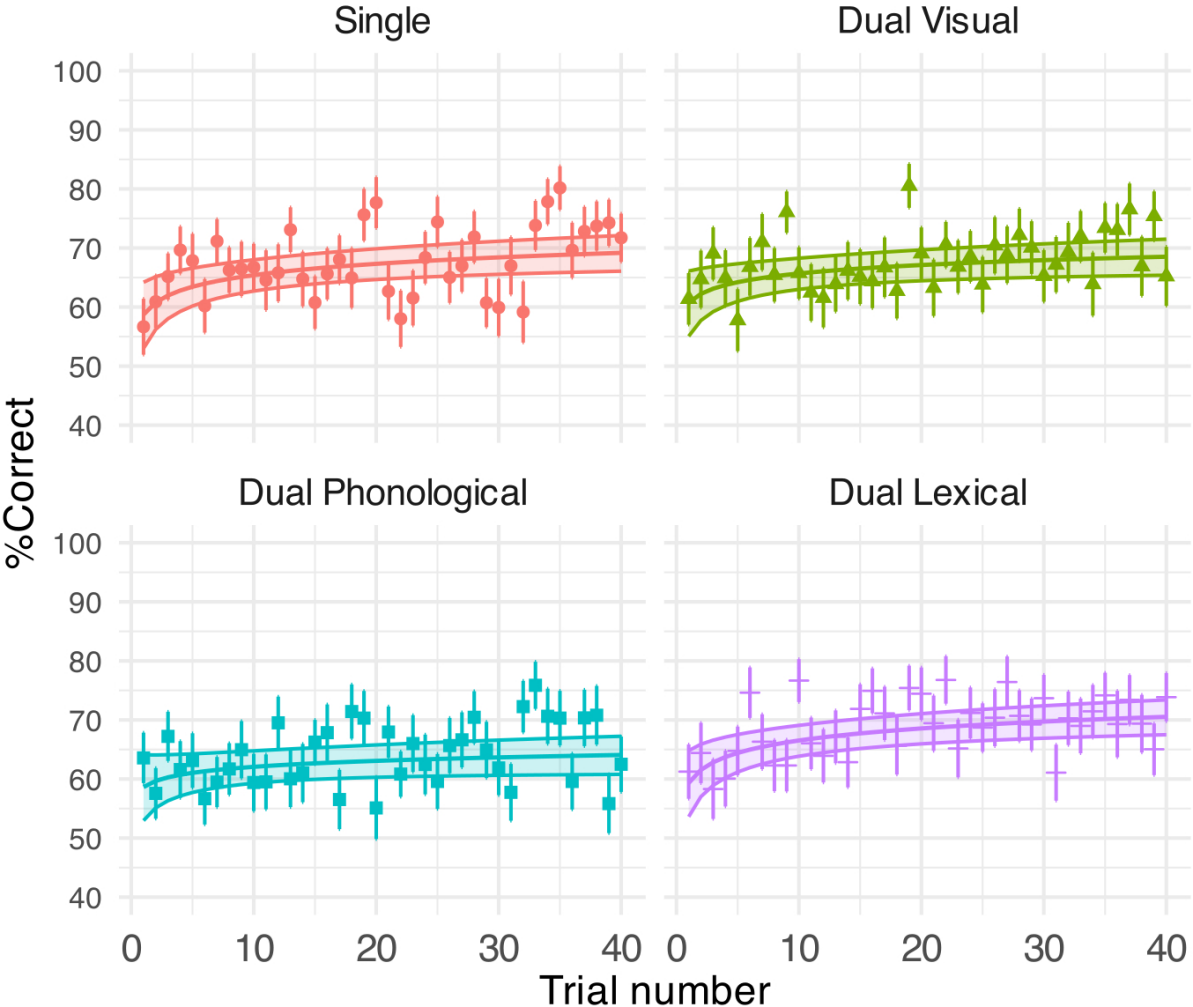
GLMM-estimated percent of correctly reported key words in speech task displayed as a function of trial (middle solid lines in the coloured areas). Each panel illustrates the results under each task condition. Filled areas represent 95% confidence intervals. Points denote the raw mean % correct obtained on each trial. Error bars indicate one SE of the mean.

Table I shows the GLMM outputs. Figure 3 shows the % correct of sentence recognition per trial per task and the GLMM predictions. The overall speech % correct were comparable under the lexical and visual secondary tasks [69% (SD = 8%) vs 68% (SD = 9%)], as well as the single speech task [68% (SD = 9.60)]. However, the speech performance was significantly lower for the phonological task than the lexical task [64% (SD = 9%) vs 69% (SD = 8%), *β* (SE) = –0.25 (0.09), *p* = 0.025, where SE is the standard error]. Trial significantly affected sentence recognition comparably in all but the phonological conditions (Table I; *p* < 0.001 for all log(trial) terms except for the nonsignificant phonological condition). Notably, the interaction between trial and task was not significant—the learning rate in phonological condition was no different than that in other conditions across the 40 trials, and participants in all conditions, thus, adapted to the dysarthric speech. Figure 3 shows that the trial effects came from a significant improvement in speech % correct over 40 trials for the single (10%), dual visual (8%), and dual lexical groups (11%) but less improvement for the dual phonological group (5%).

**TABLE I.** Model outputs for the GLMM assessing the fixed effects of task and trial on the speech task accuracy. The reference level is shown in parentheses. SE, standard error. Boldface refers to results significant at p < 0.05.

Fixed effects	*Β*	SE	*Z*	*P*
(Intercept)	0.35	0.12	2.97	**0.003**
log(trial)	0.06	0.04	1.59	0.111
Dual_lexical (dual_phonological)	0.03	0.17	0.17	0.865
Speech_single (dual_phonological)	0.00	0.17	0.02	0.985
Dual_visual (dual_phonological)	0.09	0.17	0.52	0.606
log(trial):dual_lexical (dual_phonological)	0.07	0.06	1.30	0.194
log(trial):speech_single (dual_phonological)	0.06	0.06	1.11	0.267
log(trial):dual_visual (dual_phonological)	0.03	0.06	0.56	0.579

**FIG. 4.**
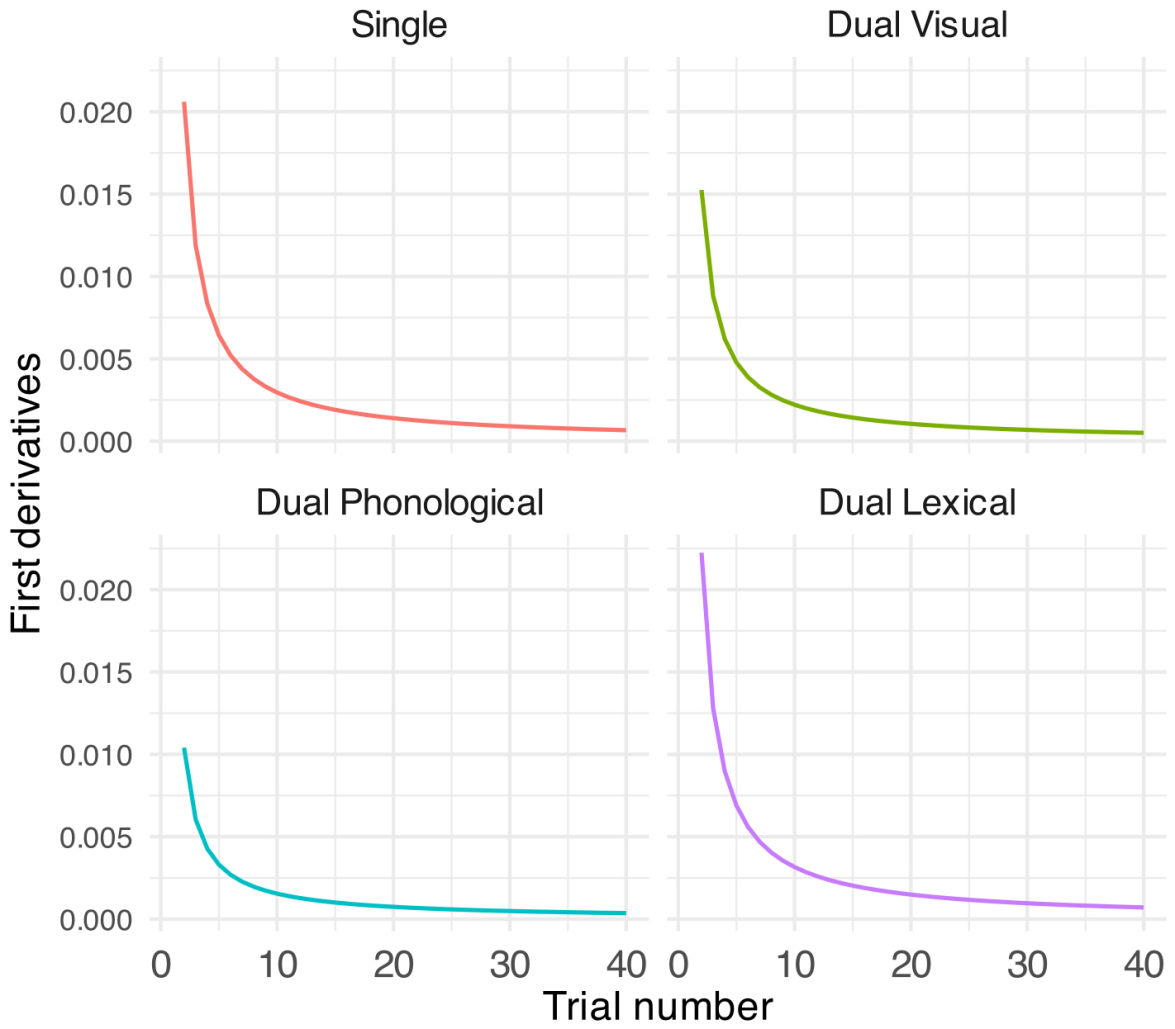
First derivatives of GLMM-estimated percent of correctly reported key words in speech task in regard to trial (solid-coloured lines). Each panel illustrates the results under each task condition.

Figure 4 further depicts the first derivatives of the GLMM predicted speech % correct regarding trial per task. The results revealed that all conditions showed fast adaptation when the task began but the rate declined quickly afterward with a very slow and plateaued adaptation rate after ten trials. The phonological condition showed a tendency of slower adaptation than the other conditions [i.e., a lower mean first derivative; single task, 0.003 (SD = 0.004); visual, 0.002 (SD = 0.003); phonological, 0.001 (SD = 0.002); and lexical, 0.003 (SD = 0.004)]. A separate GLMM on the fast adaptation phase (i.e., the first ten trials) then confirmed a significant interaction between trial and task (see Table II)—adaptation in the phonological condition was significantly slower than that in the single task and marginally slower than that in the lexical task for the first ten trials. Taken together, these results mostly support hypothesis 2—adaptation across conditions was comparable over 40 trials but was significantly suppressed in the phonological condition compared to the single condition during the fast adaptation phase.

**TABLE II.** Model outputs for the GLMM assessing the fixed effects of task and trial on the speech task accuracy for the first ten trials. The reference level is shown in parentheses. Boldface refers to results significant at p < 0.05.

Fixed effects	*B*	*SE*	*Z*	*P*
(Intercept)	0.55	0.16	3.45	**0.001**
log(trial)	−0.08	0.09	−0.88	0.377
Dual_lexical (dual_phonological)	−0.20	0.23	−0.87	0.387
Speech_single (dual_phonological)	−0.23	0.23	−1.03	0.301
Dual_visual (dual_phonological)	−0.06	0.23	−0.25	0.801
log(trial):dual_lexical (dual_phonological)	0.24	0.13	1.89	0.059
log(trial):speech_single (dual_phonological)	0.27	0.13	2.12	**0.034**
log(trial):dual_visual (dual_phonological)	0.18	0.13	1.39	0.164

### B. Performance on the dual tasks

**FIG. 5.**
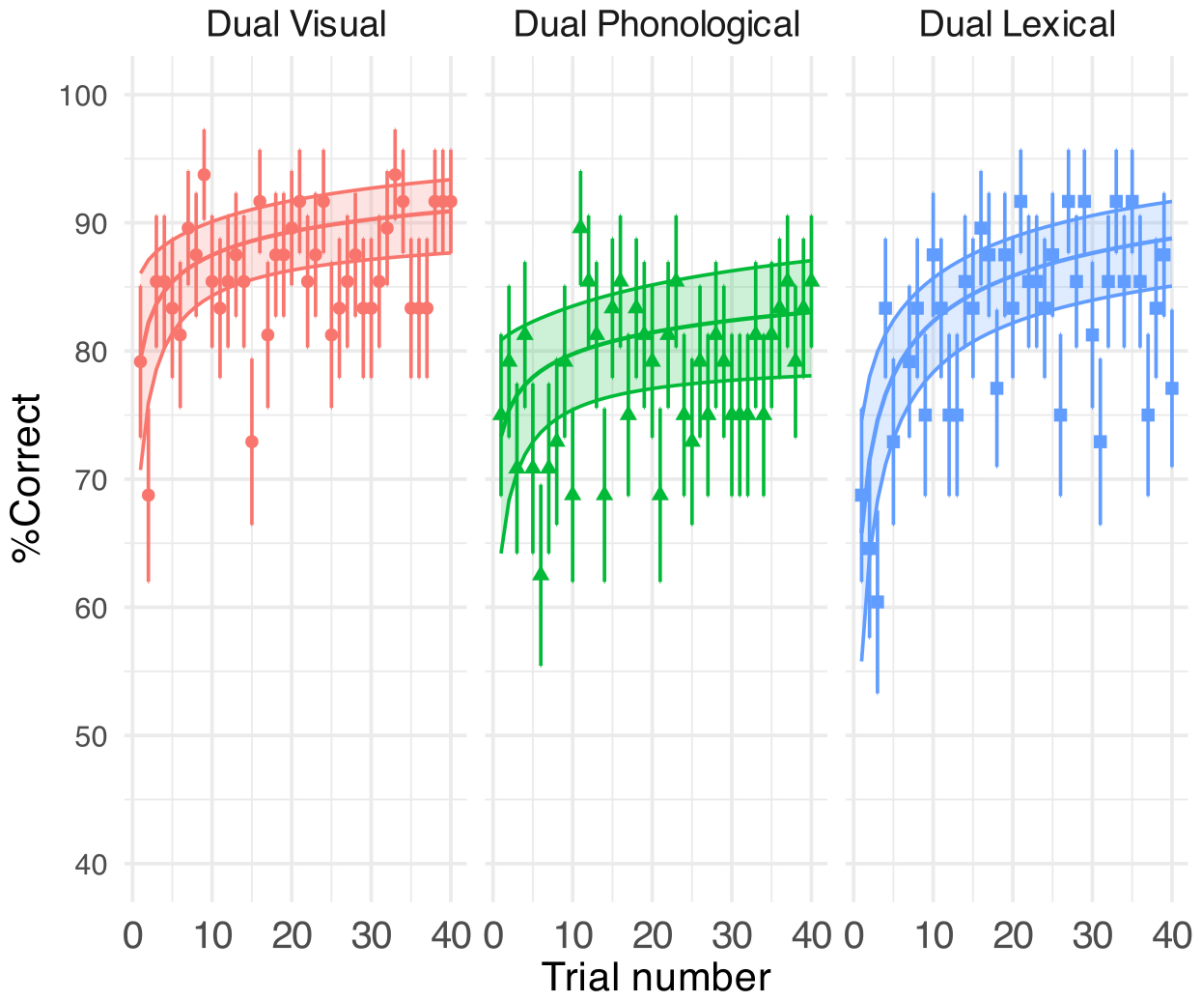
GLMM-estimated percent of correct responses for the dual task, displayed as a function of trial (middle solid lines in the coloured areas). Each panel illustrates the results under each task condition. Filled areas represent 95% confidence intervals. Points denote the raw % correct of response (i.e., number of correctly responded participants/total number of participants × 100) on each trial. Error bars indicate SE of accuracy.

**FIG. 6.**
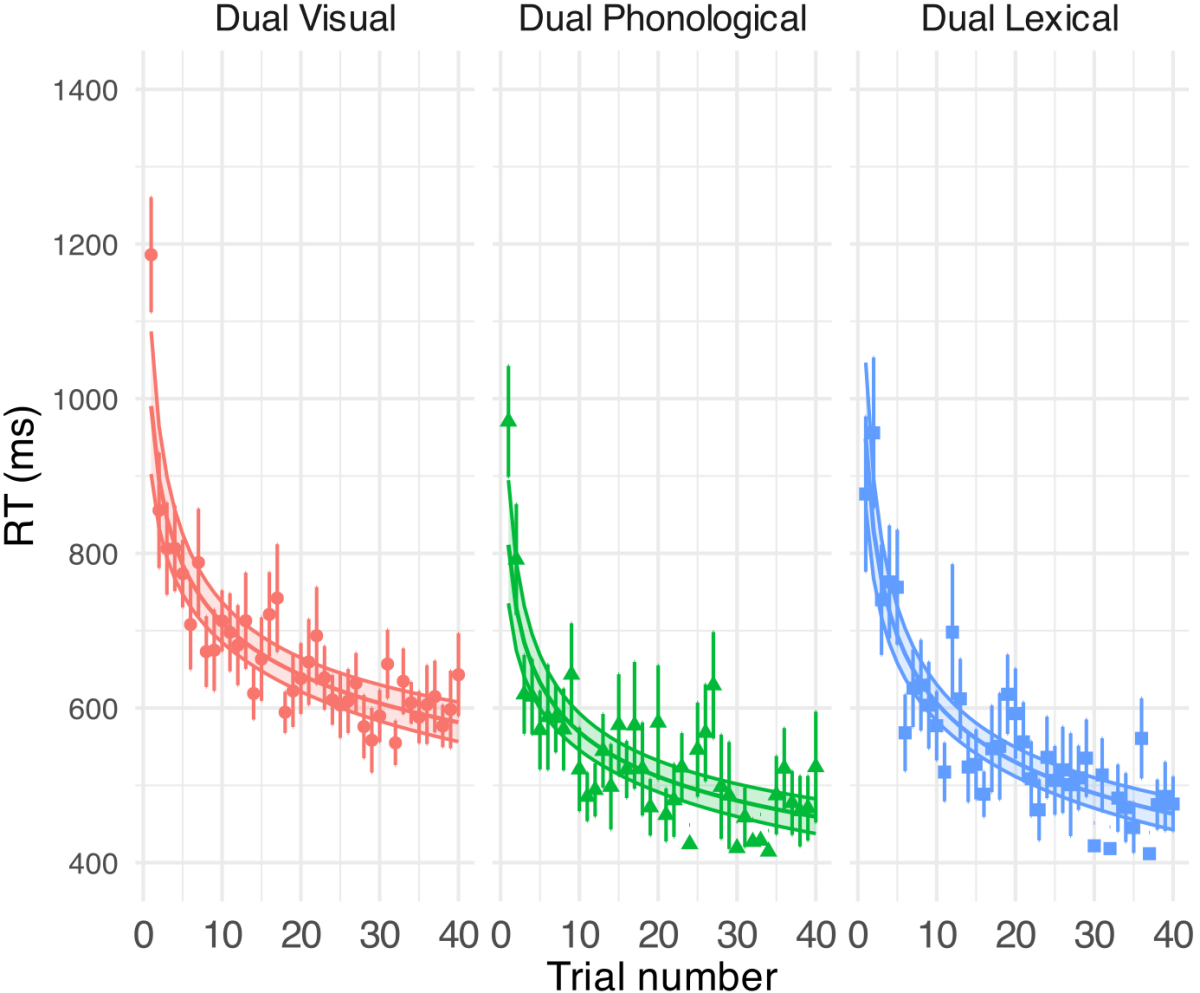
GLMM-estimated visual task RTs in milliseconds in different secondary tasks, displayed as a function of trial (middle solid lines in the coloured areas). Each panel illustrates the results under each task condition. Filled areas represent 95% confidence intervals. Points denote the raw mean RTs for correct secondary-task responses obtained on each trial. Error bars indicate SE of the mean. See Table IV for model output.

**FIG. 7.**
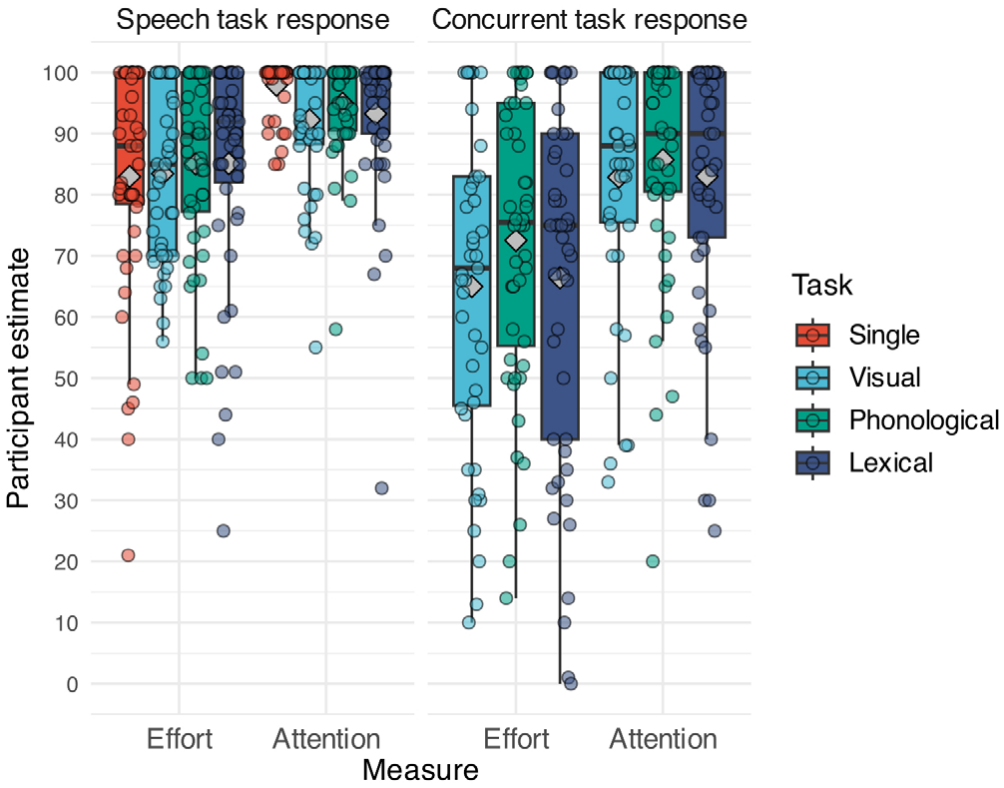
Participants' estimates on their effort and attention invested in the speech and dual tasks under different task conditions. The fill of the boxes represents task condition. Each panel shows a combination of measure and task. Points display the raw score per participant. Gray diamonds denote the group mean of each condition. See Table VI for model output.

**FIG. 8.**
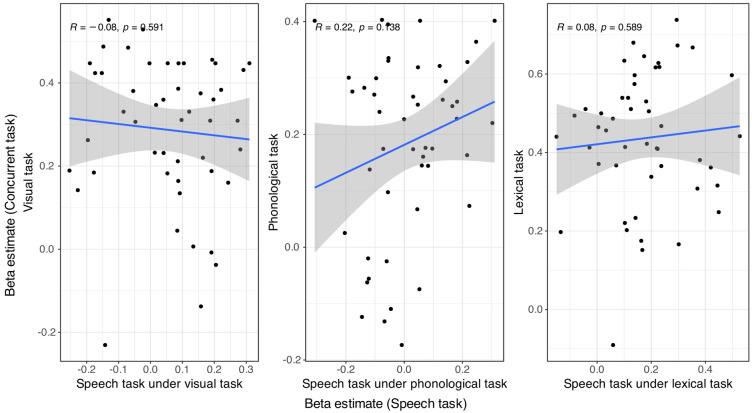
Beta estimates of the log(trial) term in the GLMMs for individual participants in different pairs of speech- and secondary-task conditions were extracted as a measure of individual slope of perceptual learning (i.e., a 1% increase in trial leads to a change in the task performance for 1% of the value of the slope). Models were fitted for the speech % correct and dual task correctness. All four models had task, log(trial), and their interaction as predictors and only included performance for the first 20 trials, where increased performances were observed for all task conditions. All models included random intercepts for participant and random slopes for trial by participant. Each panel shows a combination of speech- and secondary-task conditions. Solid blue lines display the best-fitted linear functions from individual beta estimates in the speech task conditions into their beta estimates in the secondary-task conditions. Points display the beta estimates per participant. Filled areas represent 95% confidence intervals. Pearson's correlation coefficient (*R*) and the corresponding *p* values are displayed at the top-left corner of each panel.

Figure 5 shows the response correctness (displayed in % correct) in performing the dual tasks (see Table IV in the Appendix for model outputs). Task performance was above chance (50%) in all conditions—visual [86% (SD = 10)], phonological [78% (SD = 13)], lexical [82% (SD = 11)]. Accuracy was significantly lower in the phonological than in the visual task [*β* (SE) = –0.65 (0.18), *p* = 0.001]. Accuracy significantly increased over the course of all task conditions, with the phonological task showing a marginally slower improvement than the lexical task (see Table IV). In addition, the individual slope of speech perceptual adaptation [i.e., individual beta estimate for the log(trial) term] did not predict improvements in the dual task (see Fig. 8 in the Appendix for additional details and the analysis script). See Fig. 6 for the RT results and Table V for model outputs. Analyses of the effort questionnaire are provided in Fig. 7 and Table VI in the Appendix.

## IV. DISCUSSION

This study aimed to establish the role of attentional processes in perceptual adaptation to a natural distortion, namely, dysarthric speech, with the ultimate aim to establish if adaptation is a largely automatic or controlled cognitive process. We tested whether engaging phonological cognitive processes while recognising speech dysarthric speech affected rapid perceptual adaptation. We presented participants with 40 dysarthric phrases and asked them to repeat key words while they performed 1 of 4 tasks (no task, visuomotor, lexical, or phonological task). The results showed that participants in all groups adapted to the dysarthric speech, as evidenced by increased accuracy in the speech task. Moreover, speech perception performance was reduced in the phonological group compared to the other groups, albeit by a few (but statistically significant) percent points.

Hypothesis 1 (perceptual adaptation can occur under divided attention) was, therefore, supported for the accuracy scores across all 40 trials, and hypothesis 2 (adaptation to dysarthric speech, a speech signal characterised by segmental and suprasegmental degradation, requires attention for successful adaptation) was, in part, supported as perceptual adaptation was suppressed during the fast adaptation phase for the phonological dual task condition during the first ten trials (but note that the analysis of the first ten trials was of an exploratory nature and not included in our preregistered analysis). Our results therefore largely replicate the main findings of Wang *et al.* (2023) that perceptual adaptation to variation in speech can occur under divided attention. However, our additional first derivatives analysis showed suppressed learning of the initial fast adaptation trials in the phonological group.

There are several explanations for suppressed fast adaptation in the phonological group. First, the phonological secondary task could be more difficult and this higher difficulty level affected learning in the primary speech task during the initial trials. For their experiment 1, Wang *et al.* report that manipulating the difficulty of the dual task affected perceptual adaptation in their primary speech task. Indeed, accuracy was significantly lower for the speech task under the phonological dual task than under the visual dual task (64% vs 69%, respectively) in our experiment, indicating higher difficulty of the phonological dual task. However, this indication was not borne out by the statistical analysis as there was no difference between performance on the phonological and the other two dual tasks. Second, in Wang *et al.*, perceptual adaptation was not inhibited under the phonological task for noise-vocoded speech that did not contain segmental/suprasegmental variation. Third, it could be the case that there was more adaptation in the phonological dual task compared to the other dual tasks, and this increase in adaptation might have suppressed the initial fast adaptation stage of the speech task in this condition. Yet, this possibility does not seem feasible as the phonological task showed a marginally slower improvement than the lexical task and did not differ from the visual task. Instead, it seems possible that the suppressed perceptual *fast* adaptation in the speech task under the phonological condition during the first ten trials was the result of an interaction between both tasks that resulted in a decreased performance and less adaptation compared to the lexical task. Finally, participants' subjective ratings of their attentional resources seem to support this possibility as participants estimated equally less attention to the speech task under the visual and lexical dual tasks than when performing the speech task alone (see Fig. 7 in the Appendix).

### A. Cognitive frameworks of perceptual learning/adaptation

Our results have implications for general cognitive psychology theories, in particular, for the predictive coding network (Friston and Kiebel, 2009) as these presume that attention is crucial for perceptual learning. However, the precise nature of attentional processes (focused, selective, or divided) is underspecified. Our results demonstrate that adaptation to speech with natural segmental/suprasegmental variation does not require focused or selective attention, and it is likely that this process can occur under divided attention if a long enough exposure time is considered (i.e., >10 trials). Further studies are required to establish if other types of perceptual learning or adaptation, for instance, in the visual domain, can also occur under divided attention. At the very least, based on our results and those in Wang *et al.*, these frameworks should specify the type of attentional resources that are needed for perceptual learning. Moreover, our results (across all 40 trials) with those of Wang *et al.* add to growing evidence regarding the specific nature of perceptual adaptation. It must be concluded that perceptual learning of speech is mostly automatic and can occur under cognitive load. If the concurrent task is not sufficiently engaging, largely, the same processes are required for perceptual adaptation as for the concurrent task.

### B. Limitations and future directions

We used a single phonological task, namely, syllable decision. It is unclear whether we would have found a similar result with another phonological task such as phoneme monitoring, which might engage subtly different pre-lexical processes than syllable decision. In phoneme monitoring tasks, participants listen to words (or nonwords) and decide whether the word contained a particular phoneme (e.g., does the word “penguin” contain the phoneme /p/?). As this study aimed to provide a close replication of Wang *et al.* (2023), we retained their original dual tasks, including the phonological task, but future experiments could explore whether the results replicate for other phonological dual tasks aimed specifically at segmental decisions. Moreover, the use of a phonological task does not necessarily capture all variation present in dysarthric speech, as this type of speech also includes variation at suprasegmental levels. This suprasegmental variation includes pauses, repetitions, variation in loudness and speech tempo, and variation in intonation. It is not straightforward to conceptualise a dual task that captures this type of variation, but perhaps future studies could use dual tasks that capture various aspects of these suprasegmental variations (e.g., an intonation decision task). As explained in the Introduction, we chose dysarthric speech as it was a compromise between the type of variation in the speech signal and the attentional processes required to perform the phonological dual task. Compared to other natural types of variation, such as fast speech and accented speech, dysarthric speech was considered to have fewer of the additional disadvantages associated with these other types (e.g., a timing confound or requirements for better control of the listener's accent background, respectively).

Next, it is unclear whether our finding from our exploratory analysis—that the initial fast phase of perceptual adaptation is suppressed if there is a relatively close match between the unfamiliar variation in the speech signal and the nature of the dual task—generalises to different speech processing levels. Future experiments could test this possibility directly by examining close matches between dual tasks and speech degradations/variation. For instance, a follow-up experiment could establish if adaptation to degradations or anomalies at semantic levels require lexical attentional processing. Here, participants could be asked to adapt to semantically ambiguous or anomalous sentences while performing a lexical task as also used in this experiment. Or for degradations at a lower, acoustic level, such as noise-vocoded speech, perceptual adaptation of this type of distortion could be matched with a dual task that engages basic auditory processing, such as gap detection in a noise burst, or tone discrimination.

### C. Conclusion

Our results suggest that an initial, fast phase of perceptual adaptation to dysarthric speech requires phonological attentional resources. We demonstrated that participants' perceptual learning was initially suppressed during speech recognition of phrases spoken by a speaker with dysarthria when they completed a dual task that required phonological attentional resources. Together with the results from the lexical and visual conditions, our findings demonstrate that perceptual adaptation to real-world noncanonical speech can occur under divided attention and, thus, be characterised as a largely efficient and, therefore, automatic cognitive process. This finding might inform further research into perceptual learning of speech, for instance, by explaining null effect for top-down manipulations in future studies. Moreover, our research further necessitates a reevaluation of the precise role of attentional resources in perceptual learning frameworks (Goldstone, 1998; the predictive coding theory, Friston and Kiebel, 2009; and RHT, Ahissar and Hochstein, 2004; Amitay, 2009). Our results finally suggest that if there is a close match between the type of degradation and the domain-specific attentional resource required for a dual task, the initial stage of adaptation may be suppressed to recover after further exposure.

## Data Availability

The data that support the findings of this study are available in https://github.com/hwanguc/glm_adank_etal_phonological_processing (Wang, 2025).

## Footnotes

^1^
See Gorilla.sc (Last viewed 1/30/2025).

## APPENDIX

See Tables III–VI for stimuli and model outputs, as well as Figs. 7 and 8 for the effort and attention questionnaire results and supplementary statistics, respectively.

**TABLE III.** The sentence stimuli of the speech task and the word stimuli of the dual task.

Phrase (speech task)	Word (dual task)	Session
Transcend almost betrayed	Pigeon	Practice
Mate denotes a judgement	Ambulance	Practice
Darker painted baskets	Guitar	Main
The rally found some light	Cupboard	Main
Target keeping season	Scissors	Main
Bush is chosen after	Statue	Main
Distant leaking basement	Lighthouse	Main
The wind effects the paint	Slipper	Main
Done with finest handle	Turbine	Main
Butcher in the middle	Container	Main
Career despite research	Motorbike	Main
Mistake delight for heat	Pajamas	Main
Stable wrist and load it	Sunglasses	Main
Its harmful note abounds	Trampoline	Main
Afraid beneath demand	Microphone	Main
Divide across retreat	Computer	Main
Increase a grade sedate	Detector	Main
Advance but sat appeal	Camera	Main
Technique but sent result	Basketball	Main
Kick a tad above them	Dolphin	Main
Signal breakfast pilot	Penguin	Main
Round and bad for carpet	Giraffe	Main
Admit the gear beyond	Lizard	Main
Pick a chain for action	Hamster	Main
Perceive sustained supplies	Reindeer	Main
Younger rusty viewers	Tortoise	Main
Address her meeting time	Leopard	Main
Amend estate approach	Hedgehog	Main
Had eaten junk and train	Rhino	Main
Confuse the very black	Octopus	Main
Cool the jar in private	Kangaroo	Main
Indeed a tax ascent	Jellyfish	Main
Bolder ground from justice	woodpecker	Main
Unless escape can learn	Antelope	Main
Beside a sunken bat	Ladybird	Main
To sort but fear inside	chimpanzee	Main
Thinking for the hearing	Mosquito	Main
Rode the lamp for teasing	Dragonfly	Main
Account for who could knock	Cockerel	Main

**TABLE IV.** Model outputs for the GLMM assessing the fixed effects of task and trial on the dual task accuracy. The reference level is shown in parentheses. Boldface refers to results significant at p<0.05.

Fixed effects	*Β*	SE	*Z*	*p*
(Intercept)	1.02	0.22	4.59	**<0.001**
log(trial)	0.16	0.08	2.04	**0.041**
Dual_lexical (dual_phonological)	−0.29	0.30	−0.97	0.333
Dual_visual (dual_phonological)	0.34	0.31	1.10	0.271
log(trial):dual_lexical (dual_phonological)	0.20	0.11	1.85	0.065
log(trial):dual_visual (dual_phonological)	0.10	0.11	0.91	0.363

**TABLE V.** Model outputs for the GLMM assessing the fixed effects of task and trial on the dual task RTs. The reference level is shown in parentheses. Listeners' overall RTs were significantly higher in the visual task [668.2 ms (SD = 155.0)] than in the lexical [564.2 ms (SD = 159.9)] and the phonological tasks [546.2 ms (SD = 162.6)]. Trial significantly modulated RTs in all conditions, but listeners showed markedly faster learning in the lexical than in the phonological and visual tasks. Boldface refers to results significant at p<0.05.

Fixed effects	*Β*	SE	*z*	*p*
(Intercept)	6.85	0.04	154.62	**< 0.001**
log(trial)	−0.19	0.01	−13.62	**< 0.001**
Dual_phonological (dual_lexical)	−0.16	0.06	−2.64	**0.008**
Dual_visual (dual_lexical)	0.04	0.06	0.75	0.452
log(trial):dual_phonological (dual_lexical)	0.04	0.02	2.03	**0.043**
log(trial):dual_visual (dual_lexical)	0.05	0.02	2.56	**0.011**

**TABLE VI.** Model outputs for the LM assessing effects of task condition on participants' estimated effort and attention. The reference level for the predictor is shown in parentheses. Boldface refers to results significant at p<0.05.

Model	Coefficients	*Β*	SE	*t*	*p*
Effort_speech	(Intercept)	82.98	2.45	33.86	**<0.001**
	dual lexical (single)	0.49	3.55	0.14	0.891
	dual phonological (single)	2.09	3.49	0.60	0.550
	dual visual (single)	2.13	3.47	0.61	0.540
Attention_speech	(Intercept)	97.91	1.39	70.60	**<0.001**
	dual lexical (single)	−5.68	2.01	−2.83	**0.005**
	dual phonological (single)	−2.98	1.96	−1.52	0.131
	dual visual (single)	−4.65	1.96	−2.37	**0.019**
Effort_secondary	(Intercept)	64.98	4.04	16.09	**<0.001**
	dual lexical (visual)	7.54	5.56	1.36	0.177
	dual phonological (visual)	1.44	5.56	0.26	0.796
Attention_secondary	(Intercept)	82.88	3.00	27.66	**<0.001**
	dual lexical (visual)	2.84	4.15	0.69	0.495
	dual phonological (visual)	0.14	4.15	0.03	0.974

### 1. Effort and attention questionnaire

Those were all of the 40 trials in the main task. Thank you for your participation so far! Before we send you onward to Prolific, we have a few short questions which take around 5 min to complete. Please answer all of the questions, thanks!

Question 1: Please indicate, using the slider below, how *effortful* you found it to understand the *sentences*:

Question 2 (in visual condition): Please indicate, using the slider below, how *effortful* you found it to decide the *angle of a word*:

Question 2 (in phonological condition): Please indicate, using the slider below, how *effortful* you found it to decide the *number of syllables in a word*:

Question 2 (in lexical condition): Please indicate, using the slider below, how *effortful* you found it to decide the *category of a word*:

Question 3: Please indicate, using the slider below, how much *attention* you invested on understanding the *sentences*:

Question 4 (in visual condition): Please indicate, using the slider below, how much attention you invested on deciding the *angle of a word*:

Question 4 (in phonological condition): Please indicate, using the slider below, how much attention you invested on deciding the *number of syllables in a word*:

Question 4 (in lexical condition): Please indicate, using the slider below, how much attention you invested on deciding the *category of a word*:

The data files and *R* script used for conducting formal analyses and generating figures for the experiment are available at https://github.com/hwanguc/glm_adank_etal_phonological_processing.
